# Buffering the pH of the culture medium does not extend yeast replicative lifespan

**DOI:** 10.12688/f1000research.2-216.v1

**Published:** 2013-10-15

**Authors:** Brian M Wasko, Daniel T Carr, Herman Tung, Ha Doan, Nathan Schurman, Jillian R Neault, Joey Feng, Janet Lee, Ben Zipkin, Jacob Mouser, Edward Oudanonh, Tina Nguyen, Torin Stetina, Anna Shemorry, Mekedes Lemma, Matt Kaeberlein

**Affiliations:** 1 Department of Pathology, University of Washington, Seattle, WA, 98195, USA

## Abstract

During chronological aging of budding yeast cells, the culture medium can become acidified, and this acidification limits cell survival.  As a consequence, buffering the culture medium to pH 6 significantly extends chronological life span under standard conditions in synthetic medium.  In this study, we assessed whether a similar process occurs during replicative aging of yeast cells.  We find no evidence that buffering the pH of the culture medium to pH levels either higher or lower than the initial pH of the medium is able to significantly extend replicative lifespan.  Thus, we conclude that, unlike chronological life span, replicative life span is not limited by acidification of the culture medium or by changes in the pH of the environment.

## Introduction

Aging has been studied extensively in the budding yeast *Saccharomyces cerevisiae* using two fundamentally different systems: the replicative lifespan assay and the chronological lifespan assay ^1^. Replicative life span is defined as the number of daughter cells that a mother cell can produce prior to entering an irreversible cell cycle arrest, while chronological lifespan is defined as the length of time that a yeast cell can maintain viability in a non-dividing state ^2, 3^. Numerous genetic and environmental factors have been identified that can modulate either replicative aging, or chronological aging, or both.

Replicative aging has been studied almost exclusively by maintaining individual cells on the surface of a nutrient agar plate, microdissecting daughter cells away from the mother cells, and counting the number of daughter cells that the mother cell produces prior to senescence ^4^. Generally, rich YPD medium (2% glucose) is used for replicative lifespan assays. Calorie restriction by reducing the glucose concentration of the medium to 0.5% or lower has been shown in numerous studies to extend lifespan in different wild type strain backgrounds between 10–40% ^5, 6^.

Several methods have been described for studying chronological aging. The most widely utilized protocol involves culturing yeast cells in synthetic complete liquid medium with 2% glucose as the carbon source, either under shaking or static conditions, in culture tubes or 96-well plates ^7, 8^. Alternative, but less frequently used, liquid culture methods for chronological aging involve culturing cells in rich YPD medium, using a respiratory carbon source such as glycerol, or transferring cells to water once they have reached stationary phase growth arrest ^9, 10^. A plate-based assay for chronological life span analysis has also been described in which cells are growth arrested through limitation for tryptophan ^11^. In all of these assays, viability over time is determined by restoring a small subset of the population to nutrient rich growth conditions and assaying their ability to re-enter the cell cycle, either through quantification of colony forming units on solid-agar plates or through outgrowth kinetics in liquid culture ^3, 12^. Similar to the case for replicative lifespan, calorie restriction by reducing the initial glucose concentration of the culture medium can extend chronological lifespan, generally by more than 100% ^13, 14^.

One important feature of the standard method for determining chronological aging is that the culture medium becomes acidified over the first few days of the experiment, with pH dropping from an initial value of around 4.0 to 2.5–2.9 within 96 hours ^15^. This acidification of the external environment results from the production of organic acids, including acetic acid, following fermentation of glucose to ethanol and subsequent utilization of ethanol as a carbon source once the glucose is depleted. Preventing medium acidification by buffering the culture to a pH of 6.0 with either citrate phosphate buffer or low salt MES buffer results in a more than doubling of chronological lifespan ^15^. Calorie restriction, or switching the yeast culture to a non-fermentable carbon source, such as glycerol or ethanol, also prevents acidification and results in a similar magnitude of chronological lifespan extension as buffering ^15, 16^.

Although the two yeast aging assays are nearly always studied independently, it is clear that they share at least some overlap. As mentioned above, calorie restriction extends both replicative and chronological lifespan, as do a few genetic interventions, such as deletion of either *TOR1* or *SCH9*, both of which are nutrient-responsive kinases ^7, 17– 19^. In addition, it has been shown that chronologically aged cells have reduced replicative lifespan when returned to rich growth conditions ^9^. This reduction in replicative lifespan following chronological aging appears to be mediated through changes in mitochondrial function, since the chronologically old cells that retain the lowest mitochondrial membrane potential also have the longest replicative lifespan following resumption of cell division ^20^. Calorie restriction or buffering the culture medium of the cells during chronological aging also protects against subsequent replicative lifespan reduction ^21^, raising the possibility that medium acidification directly influences both types of yeast aging. To assess this possibility, we performed replicative lifespan analysis on wild type BY4742 mother cells under either standard conditions or on rich media buffered to different pH values. We were unable to detect a significant replicative lifespan extension from buffering the culture medium under any of the conditions examined, including those conditions that robustly extend chronological lifespan.

## Methods

### Replicative lifespans

All lifespan experiments were performed in the BY4742 strain background (Thermo Scientific, Waltham, MA) as previously described ^4, 22^. Virgin daughter cells were isolated and allowed to grow into mother cells while their corresponding daughters were microdissected using Zeiss Axioskop 40 dissection microscopes and manually counted until the mother cell could no longer divide ^4^. YEP agar plates (1% yeast extract, 2% bacto-peptone, 2% agar) containing 2% glucose (YPD) were utilized and strains were grown at 30°C during the day, dissected at room temperature, and placed in a refrigerator at 4°C over night. Daughter cells were removed from each mother cell roughly every 2 hours by micromanipulation ^4^. Cells were scored as senescent when they had failed to divide for at least eight hours of incubation at 30°C. Terminal morphology was defined as the budded state of the mother cells upon senescence ^23^. All experiments were performed by a team of dissectors who were blinded to the identity of the strains under examination in any given experiment. Prism Graphpad 5.0 was used for data analysis. Statistical significance for differences in median lifespan was determined using the Wilcoxon Rank-Sum test. Budded and unbudded states were determined visually for each mother cell assayed and statistical comparisons of budding rates utilized Fischer’s Exact two-tailed test. Multiple comparison corrections were performed using the Bonferroni correction.

### Preparation of media

Stock buffers were prepared at 1 M in deionized water and pH was adjusted by addition of appropriate molar ratios of conjugate acid and conjugate base, or by empirical adjustment with HCl or NaOH. Buffer reagents were obtained from Sigma-Aldrich (St Louis, MO). The following buffers were used: Tris(hydroxymethyl)aminomethane (Tris) (; 3-(N-morpholino)propanesulfonic acid (MOPS); 2-(N-morpholino)ethanesulfonic acid (MES); citrate buffer (sodium citrate and citric acid); acetate buffer (sodium acetate and acetic acid). Stock buffers were sterilized by filtration using a VWR International syringe driven 0.2 micron cellulose acetate membrane filter. Buffers were diluted to 100 mM final concentration in YPD agar after autoclaving and cooling to ~55°C. Appropriate stock buffer pH was empirically determined, as necessary to adjust the pH of YPD liquid media at room temperature to the indicated final pH. Measurement of pH was performed using an Accumet Excel XL15 pH meter. The pH of agar media was further verified by use of EMD colorpHast pH-indicator strips. For adjustment of media pH without buffer addition, the indicated acid or base was added to achieve the desired pH prior to autoclaving.

## Results

### pH and buffering

**Figure 1.**
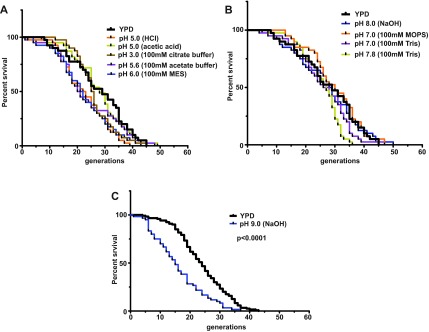
Replicative lifespans with pH adjusted to 7–8 by buffering or NaOH ( **A**), or pH 6.0 or below by acids or buffers ( **B**) and to pH 9.0 by NaOH ( **C**).

Lowering the pH of YPD agar plates to 5.0 by HCl (p = 0.0218) and to 6.0 using MES buffer (p = 0.0165) trended toward a decrease in lifespan that was statistically significant without adjusting for multiple comparisons (α = 0.05), but which did not reach significance after adjusting for multiple comparisons using the Bonferroni correction (α = 0.0056) ( Figure 1A, Table 1). Lowering pH to 5.0 by acetic acid (p = 0.8869) or buffering to pH 5.6 using acetate buffer (p = 0.0896) had no detectable effect on lifespan. Further reduction of pH to 3.0 using a citrate buffer (p = 0.3412) also had no effect on lifespan.

**Table 1.** Effects of pH and buffering on terminal morphology and statistical analysis for experimentally matched RLS data shown in Figure 1.

pH	Buffer or pH adjusted by	Median RLS	RLS vs. YPD P	Budded	Unbudded	%B	RLS B	%U	RLS U	B&U vs. YPD P value
8.0	NaOH	28.5	0.7658	18	22	45.0	23	55.0	30.5	0.3711
7.8	Tris buffer	27	0.1754	23	17	57.5	27	42.5	26	1
7.0	MOPS buffer	30	0.4161	21	19	52.5	25	47.5	36	0.8224
7.0	Tris buffer	27	0.3653	21	18	53.8	25	46.2	28	0.8224
6.8	YPD	29	-	23	17	57.5	25	42.5	34	-
6.0	MES buffer	21.5	0.0165	13	27	32.5	16	67.5	22	0.0424
5.6	Acetate buffer	20.5	0.0896	15	25	37.5	18	62.5	25	0.1165
5.0	HCl	22.5	0.0218	11	29	27.5	18	72.5	23	0.0123
5.0	Acetic acid	28	0.8869	14	25	35.9	25	64.1	30	0.0722
3.0	Citrate buffer	25.5	0.3412	11	29	27.5	26	72.5	25	0.0123

RLS, median replicative lifespan; U, unbudded; B, budded.

Buffering YPD media to pH 7 by MOPS (p = 0.4161) or Tris (p = 0.3653) had no detectable effect on yeast lifespan ( Figure 1B). Increasing the pH of YPD to 8.0 by NaOH (p = 0.7658) or buffering at pH 7.8 with 100 mM Tris (p = 0.1754) also has no detectable effect on lifespan. Raising pH further to 9.0 by sodium hydroxide (p < 0.0001) resulted in a significant reduction of lifespan ( Figure 1C), and cells on YPD buffered to pH 9.0 with 100 mM Tris buffer did not divide and, thus, replicative lifespan could not be determined for this condition.

### Terminal morphology

Terminal morphology is defined as the budded state of the mother cells upon senescence ^23^. Terminal morphology frequency was not significantly altered when pH was buffered at 7.0 by MOPS (p = 0.8224) or 7.0 (p = 0.8224) or 7.8 by Tris (p = 1) or by adjustment of pH to 8.0 by sodium hydroxide (p = 0.3711). Manipulations that lowered the pH displayed a trend toward a higher percentage of unbudded cells upon arrest ( Table 1), but this did not reach statistical significance after correcting for multiple testing (α = 0.0056).

Yeast replicative lifespan and terminal morphology in culture media of varying pH The replicative lifespan of yeast (BY4742 strain background) cultured in media of varying pH. Column L (“lifespans”; LS) through AE represent the number of daughter cells produced by each mother cell and the endCode (column AF-AY) represents the terminal morphology of the corresponding numbered mother cell. End lost represents the number of cells that did not receive a terminal cell morphology designation. U = unbudded, S = small bud (<50% size of mother), L = large bud (>=50% size of mother), C = cluster of cells.

## Discussion

The results presented here demonstrate that, unlike chronological lifespan, acidification of the culture medium does not limit replicative lifespan under standard conditions. This is relevant information, because it rules out the possibility that interventions shown to extend replicative lifespan are acting by either reducing the production and secretion of organic acids into the environment or by increasing resistance to acid stress.

The biological relevance of acidification limiting chronological lifespan has been an area of contention within the field, due in part to concerns that cell death due to acidification may be a yeast specific phenomenon ^1^. Evidence supporting this concern has been provided by parallel analyses of replicative and chronological lifespan for yeast deletion mutants corresponding to *Caenorhabditis elegans* genes that increase lifespan when their expression is reduced. A significant enrichment for long replicative lifespan was found among this set of yeast deletions ^24^, but no enrichment for increased chronological lifespan under standard conditions was observed ^25^. On the other hand, there is evidence that a similar acid-induced mechanism of senescence occurs in mammalian cells, at least in culture, suggesting the possibility that the intracellular response to external pH may be conserved ^26, 27^.

The trend toward reduced lifespan noted under some of the conditions tested is of interest and may warrant further study. The significant reduction in lifespan associated with adjusting to pH 9.0 by NaOH may reflect a reduced ability of yeast to proliferate under basic conditions, which is consistent with the inability of yeast cells to grow in the replicative lifespan assay when the YPD was buffered to pH 9.0 by Tris buffer. Among the acidic conditions tested, any effects on lifespan are likely to be due to the composition of the buffer rather than a direct result of the lower pH. As evidence for this, we note that YPD buffered to pH 3.0, the most acidic condition tested, had no effect on lifespan.

In summary, we find no evidence that acidification of the culture medium, or pH changes in general, limit replicative lifespan in the BY4742 laboratory yeast strain under standard conditions. Buffer conditions that dramatically extend chronological lifespan of this strain do not similarly extend replicative lifespan. These data demonstrate that effects of acidification on aging in yeast are likely to be restricted to non-dividing cells.
