# Supplementary material for: Buffering the pH of the culture medium does not extend yeast replicative lifespan
Source: F1000Res. 2013 Oct 15;2:216. [Version 1] doi: 10.12688/f1000research.2-216.v1 (PMC3886788; doi:10.12688/f1000research.2-216.v1)
Supplement: Peer review history

# Peer review: Referee response for version 1

**Published:** 2013-12-03

**Authors:** Cory Dunn

**Affiliations:** 1 Department of Molecular Biology and Genetics, Koç University, Istanbul, Turkey

In this work, Wasko *et al.* investigate whether altering the pH of rich medium can result in changes to the replicative lifespan of *Saccharomyces cerevisiae* strain BY4742. They find that replicative lifespan, except in the most extreme case of pH 9.0, is not affected by extracellular pH. Their results indicate that mutations and conditions increasing replicative lifespan are unlikely to do so through altering acidity/alkalinity of rich medium.

Recently, it was demonstrated by Hughes *et al*. *Nature* 492:261-265 that deletion of the vacuolar V _1_V _O_-ATPase subunit Vma2p, which results in an increased vacuolar pH, causes a drastic shortening of replicative lifespan. Also, Hughes *et al*. found, by a qualitative microscopy assay, that over expression of *VMA1* or *VPH2 *decreased vacuolar pH and that replicative lifespan was coincidentally increased.

Extracellular pH has been demonstrated to impinge on vacuolar pH, more prominently in the case of increased medium pH. This has been examined most comprehensively by Brett *et al*. *PLoS ONE* 6(3):e17619, but has also been demonstrated by others using genetic, fluorophore-based, and even ^31^P-NMR-based assays. If extracellular pH impinges upon vacuolar pH, and if replicative lifespan depends upon vacuolar pH, then why is replicative lifespan not changed upon altering extracellular pH? A discussion of this point is definitely warranted in a revised version of this manuscript.

I have read this submission. I believe that I have an appropriate level of expertise to confirm that it is of an acceptable scientific standard.

# Peer review: Referee response for version 1

**Published:** 2013-10-22

**Authors:** Karim Mekhail

**Affiliations:** 1 Department of Laboratory Medicine and Pathobiology, University of Toronto, Toronto, Canada

Yeast cells have been successfully used for decades to identify conserved genetic and environmental factors that control cellular lifespan, which often underlies organismal lifespan. In yeast, the lifespan of dividing and non-dividing cells can be assessed via measurement of replicative and chronological lifespan, respectively. It was previously shown that chronological lifespan can be optimized through appropriate buffering of the cell culture media. In this brief *F1000Research* Research Article, Wasko *et al.,* investigate whether replicative lifespan is also affected by environmental pH levels. The authors conduct a series of technically sound and straightforward experiments to address this question. Micromanipulation of BY4742 cells cultured under various pH conditions did not reveal any significant relationship between extracellular pH levels and replicative lifespan. The authors were quite careful in their interpretation of the findings and appropriately acknowledged limitations. For example, possible effects related to the different types of buffering agents used were noted. In addition, clear statements highlighting that the results of this study are obtained via reliance on a single yeast strain (BY4742) were included throughout the results and discussion sections. Moreover, statistical analyses were generally appropriate as presented. Overall, as suggested by the authors, the presented data suggest that the effects of acidification on aging in yeast are likely to be restricted to non-dividing cells. I only make the minor suggestion to consider adding the word “BY4742” before the word “yeast” in the title.

I have read this submission. I believe that I have an appropriate level of expertise to confirm that it is of an acceptable scientific standard.
